# Clinical Update of the Latest Evidence for CardioMEMS Pulmonary Artery Pressure Monitoring in Patients with Chronic Heart Failure: A Promising System for Remote Heart Failure Care

**DOI:** 10.3390/s21072335

**Published:** 2021-03-27

**Authors:** Jasper J. Brugts, Sumant P. Radhoe, Dilan Aydin, Dominic A. Theuns, Jesse F. Veenis

**Affiliations:** Thorax Center, Department of Cardiology, Erasmus MC, University Medical Center Rotterdam, 3015 GD Rotterdam, The Netherlands; s.radhoe@erasmusmc.nl (S.P.R.); d.aydin@erasmusmc.nl (D.A.); d.theuns@erasmusmc.nl (D.A.T.); j.veenis@erasmusmc.nl (J.F.V.)

**Keywords:** CardioMEMS, telemonitoring, e-health, remote care, heart failure, sensor

## Abstract

The CardioMEMS pulmonary artery (PA) monitoring system placed in the left lower lobe pulmonary artery is capable of measuring pulmonary artery pressure remotely as a surrogate of intracardiac filling pressures and volume status. The technique is safe and reliable. By using remote PA monitoring for proactive medical interventions, there is a growing body of clinical evidence for a substantial, robust reduction in HF hospitalizations in various populations (clinical trial setting, post-marketing studies and real-world experiences). This review summarizes the clinical evidence, outlines future perspectives, and aims for remote patient care in heart failure using CardioMEMS.

## 1. Introduction

In the last ten years, the field of heart failure has changed quite drastically. Several drugs have been proven more effective in improving prognosis of heart failure (HF) patients [1]. Device therapy and biventricular pacing is part of standard heart failure care and expanding. Still, the prevalence and incidence of heart failure are rising worldwide. This is related to the aging of the population, increasing risk factors for cardiovascular disease like obesity and the success of primary percutaneous coronary intervention (PCI) by which patients survive their myocardial infarction. For Western-World countries, the chronic HF population compromises about 1–2% of the general population [1]. In all countries, the burden on hospitals for heart failure care is enormous as HF admissions, patient contacts and re-admissions are very frequent. In order to cope with these developments, remote care in HF is essential. The current COVID-19 pandemic might even have improved the understanding in many healthcare systems that remote care is the way forward, with the aim and focus to deliver care outside the hospital to relieve the scarce resources at hand. Telemonitoring and eHealth solutions are an important step to achieve these goals of remote patient care in HF. Many attempts with simple non-invasive tools of telemonitoring have been studied but proven of only small benefit with inconsistencies in literature. Still, non-invasive tools such as remote monitoring of weight or electrocardiogram (ECG) or symptoms by telephone is of low cost and available to large groups of (less symptomatic) patients with chronic HF [2]. Therefore, non-invasive telemonitoring tools are important to study and can have a major impact on large groups.

Invasive remote monitoring tools are available in HF care and consist of specially developed sensors. One of the holy grails for HF therapy would be to measure filling pressures remotely in a patient with heart failure. The majority of HF drugs, such as diuretics and vasodilators, aim to lower filling pressures. It has been shown that rising filling pressures are the first hemodynamic sign of an upcoming period of decompensation. The rise in filling pressure comes well before the signs of overt clinical congestion [3]. This could provide an approach for early proactive interventions based upon filling pressures in a patient outside the hospital with the central aim to prevent hospitalization. The CardioMEMS HF system uses a specially developed sensor placed in the left lower lobe pulmonary artery (PA), and is able to measure PA pressures remotely as a surrogate for volume status [4,5].

This review will provide an overview of the clinical evidence for the CardioMEMS HF monitoring system in patients with chronic heart failure.

## 2. The CardioMEMS HF System

**Figure 1:**
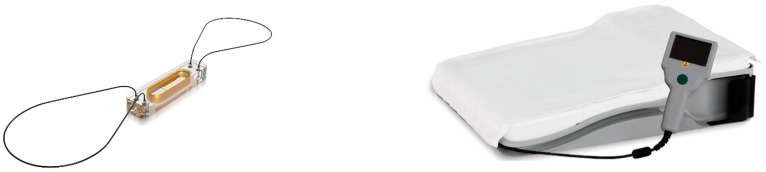
The CardioMEMS pulmonary artery pressure sensor and patient electronics unit for daily pressure measurements. Figures used with permission from Abbott (Abbott, Sylmar, CA, USA).

The CardioMEMS HF system consists of an implantable wireless sensor, a patient and hospital electronics system and a patient database (Integrated Merlin.net secure website for patient data management). The sensor measures PA pressures using microelectromechanical system technology with a piezoelectrical membrane (wireless) (Figure 1) [1,2,3,4,5]. Distortion of the piezoelectrical membrane in the sensor (in the vessel) changes the resonance frequency signal which corresponds to a pressure shift. The sensor is implanted in a branch of the left pulmonary artery through the femoral vein. CardioMEMS is the only invasive heart failure (HF) remote monitoring sensor with Food and Drug Administration (FDA) approval and European Conformity (CE) mark. The CardioMEMS HF system is recommended in the European Society of Cardiology (ESC) guidelines of 2016 with a class II b level B recommendation, which is expected to be upgraded in the newest version of the guidelines in 2021 with evidence from several new studies [1,6,7,8,9,10].

The CardioMEMS PA sensor has no internal power supply and is fully compatible with implantable cardioverter defibrillators (ICDs), (cardiac resynchronization therapy) CRT devices and left ventricular assist devices (LVADs). Furthermore, the sensor is MRI (magnetic resonance imaging) compatible. Potential complications are mostly related to the implant procedure, which is of very low risk as will be discussed extensively in this review. Patients who are already on anticoagulants are restarted on treatment after sensor implantation. Patients who are not yet receiving anticoagulants should start treatment with aspirin (81 mg/day or 325 mg/day) and clopidogrel (75 mg/day) concomitantly for 1 month after implant, after which aspirin monotherapy is to be continued lifelong [4]. The need for anticoagulants can potentially cause adverse (bleeding) events. The sensor was developed to last lifelong, but several problems have been encountered. A case report has described dampened PA pressure waveforms months after successful implantation [11]. In this patient, dampening of the signal was most likely caused by sensor migration and was resolved by recalibration. Another case report has described sensor migration, which may occur during the implant procedure, but also later onwards during the monitoring phase. In this case, no adverse outcomes occurred and the sensor was successfully recalibrated with repeated right heart catheterization and could therefore still be used to manage HF therapy [12]. Currently, there is no detailed information on pressure range limitations, but one study investigated CardioMEMS in patients suffering from pulmonary arterial hypertension (PAH) with systolic PA pressures over 90 mmHg, and found the device to be feasible and safe, which is promising for the target HF population [13].

This article provides an overview of the latest promising information on using CardioMEMS for telemonitoring of heart failure in New York Heart Association (NYHA) class III patients. The increasing body of evidence for this technique suggests exciting opportunities to improve future heart failure management.

## 3. Current Evidence: Overview of Studies

Currently, multiple studies have investigated the safety and clinical efficacy of the CardioMEMS PA sensor in chronic HF patients. All studies are discussed in detail below, and an overview of the study characteristics and their results is provided.

### CHAMPION Trial 

**Figure 2:**
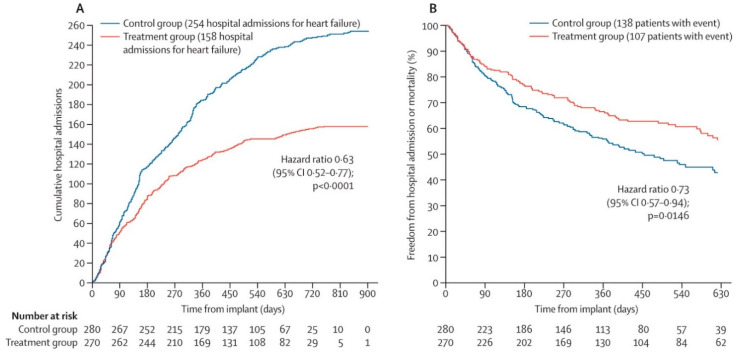
Short-term results from the CHAMPION trial: Cumulative heart-failure-related hospitalizations during entire period of randomized single-blind follow-up (**A**), and freedom from first heart-failure-related hospitalization or mortality during the entire period of randomized follow-up (**B**). Reprinted from The Lancet Volume 377, Issue 9766, Abraham et al., Wireless pulmonary artery hemodynamic monitoring in chronic heart failure: a randomized controlled trial, pages 658–666, Copyright 2011, with permission from Elsevier [4].

The CardioMEMS Heart Sensor Allows Monitoring of Pressure to Improve Outcomes in NYHA Class III Heart Failure Patients (CHAMPION) trial, published in 2011, was the first clinical trial confirming that implantable hemodynamic monitoring systems can reduce the number of HF hospitalizations as a landmark study [4]. The CHAMPION trial studied 550 patients with chronic HF in NYHA functional class III with 1 previous HF admission in the past 12 months from 64 participating centers in the USA. The patients were randomized to the CardioMEMS HF system (n = 270) versus the control group (n = 280) and were studied for at least 6 months [4]. All patients received a CardioMEMS device implant, and were instructed to perform daily pressure readings. In the CardioMEMS HF system group, the treating physicians used the daily measurement of pulmonary artery pressures in addition to standard care with the aim to normalize PA pressures and proactively react upon significant rises in PA pressures. In the control group, treating physicians had no information on PA pressures. The primary efficacy endpoint was the rate of HF hospitalization at 6 months. The primary safety endpoint was the freedom of device- or system-related complications (DSRC) and freedom of sensor failures at 6 months. The mean age of these patients was 61 years and 73% were men. The majority of patients suffered from heart failure with a reduced ejection fraction (HFrEF), 78% with a left ventricular ejection fraction (LVEF) < 40%. On baseline, 90% of all patients received a beta-blocker, 78% a RAS-inhibitor and 42% a mineralocorticoid receptor antagonist (MRA) [4]. In 6 months, 120 HF-related hospitalizations occurred in the control group (rate 0.44) and 84 in the CardioMEMS group (0.32) which resulted in a significant reduction in HF hospitalizations of 28% (HR 0.72 95% CI 0.60–0.85; *p* < 0.0001). During the entire follow-up of 15 months, the treatment group had a 37% reduction in HF-related hospitalizations as compared to the control group (Figure 2). Considering safety, the freedom of DSRC was 98.6% and freedom of sensor failures 100%. Clearly, the CHAMPION trial established that additional hemodynamic information is superior to clinical signs and symptoms only and allows for improved HF management remotely [4].

## 4. Open-Access Extension of the CHAMPION Trial 

**Figure 3:**
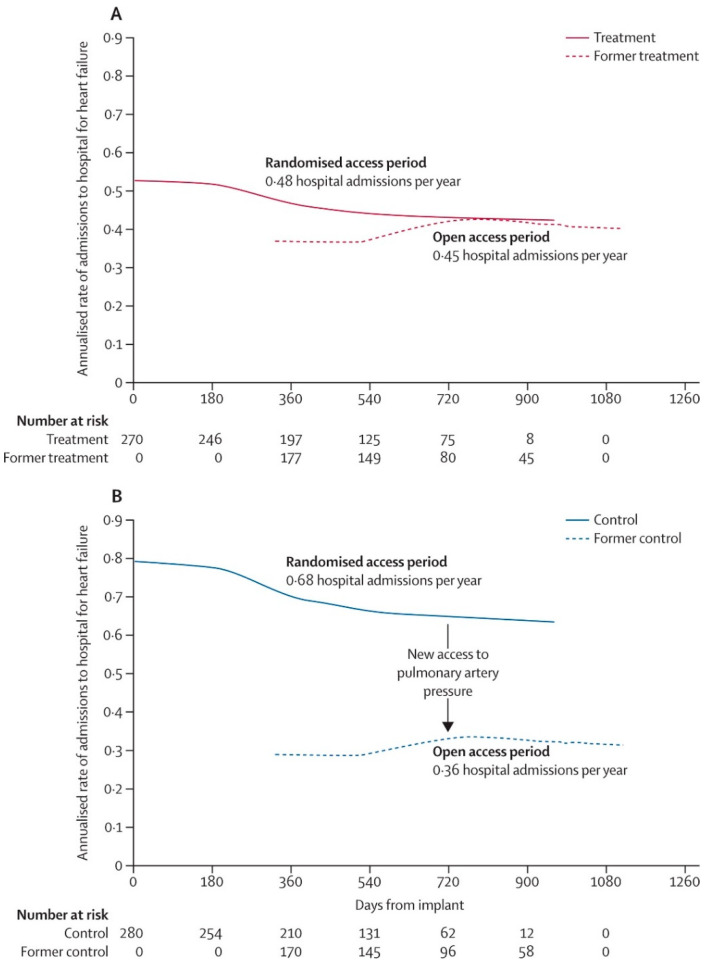
Long-term results from the CHAMPION trial: Effect of continued access to pulmonary artery pressure information on the change in rates of admission to hospital for heart failure during open access in the former treatment group (**A**) and on the change in rates of admission to hospital for heart failure during open access and in the former control group (**B**). Reprinted from The Lancet, Volume 387, Issue 10017, Abraham et al., Sustained efficacy of pulmonary artery pressure to guide adjustment of chronic heart failure therapy: complete follow-up results from the CHAMPION randomized trial, pages 453–461, Copyright 2016, with permission from Elsevier [5].

**Figure 4:**
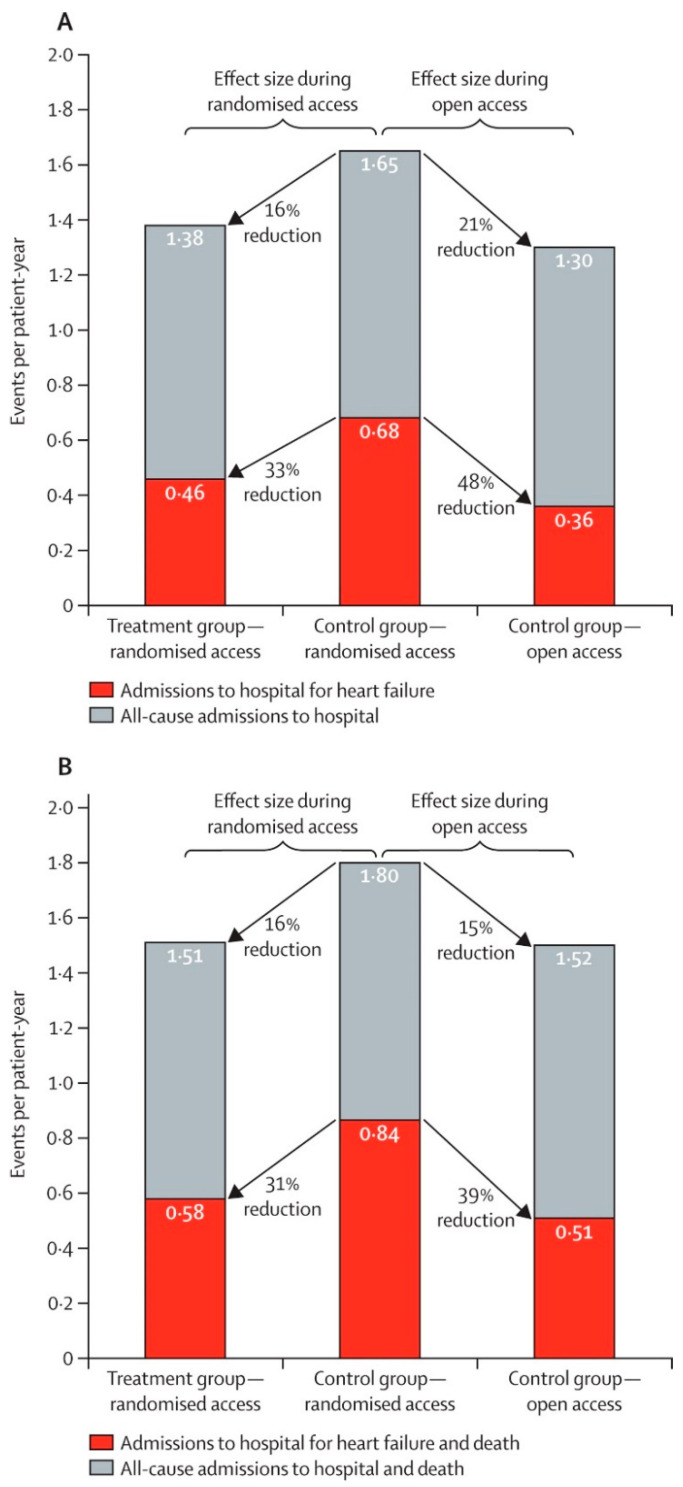
Long-term results from the CHAMPION trial: Effect of pulmonary artery pressure-guided heart failure management on rates of admission to hospital (**A**) and on combined rates of admission to hospital and mortality (**B**). Reprinted from The Lancet, Volume 387, Issue 10017, Abraham et al., Sustained efficacy of pulmonary artery pressure to guide adjustment of chronic heart failure therapy: complete follow-up results from the CHAMPION randomized trial, pages 453–461, Copyright 2016, with permission from Elsevier [5].

After the randomized access period, an open-access period in the CHAMPION trial was extended where former control group patients were also managed by using PA pressure feedback [5]. After pulmonary artery pressure information became available to guide therapy during open access (mean 13 months), rates of admissions to hospital for heart failure in the former control group were reduced by 48% (HR 0.52 [95% CI 0.40–0.69]; *p* < 0.0001) compared to admission rates during randomized access which confirms the demonstrated benefit of the CardioMEMS HF system monitoring (Figure 3 and Figure 4). Furthermore, over the complete randomized follow-up period averaging 18 months, heart failure admission rates were significantly lower in the treatment group as compared to the control group (HR 0.67 [95% CI 0.55–0.80]; *p* < 0.0001). These extended follow-up findings are important as they confirm the significant long-term benefit of remote monitoring by the CardioMEMS HF system with even slightly more pronounced effects. The authors showed that the medication changes over time were mainly related to changes in diuretic dose and vasodilators [5].

### 4.1. MEMS-HF 

**Figure 5:**
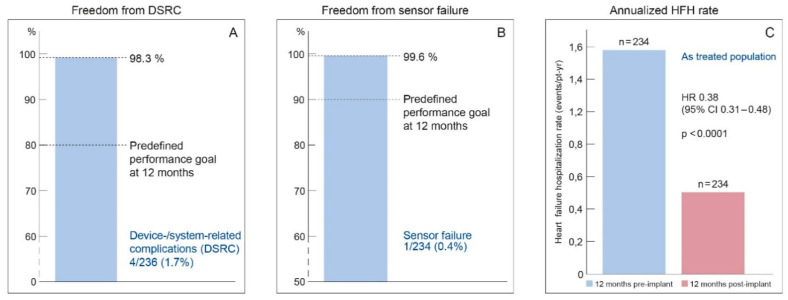
Annualized heart-failure-related hospitalization rates pre-implant and post-implant as reported in the MEMS-HF study. Reprinted from the European Journal of Heart Failure, Volume 22, Issue 10, Angermann et al., Pulmonary artery pressure-guided therapy in ambulatory patients with symptomatic heart failure: the CardioMEMS European Monitoring Study for Heart Failure (MEMS-HF), pages 1891–1901, with permission from Wiley [6].

**Figure 6:**
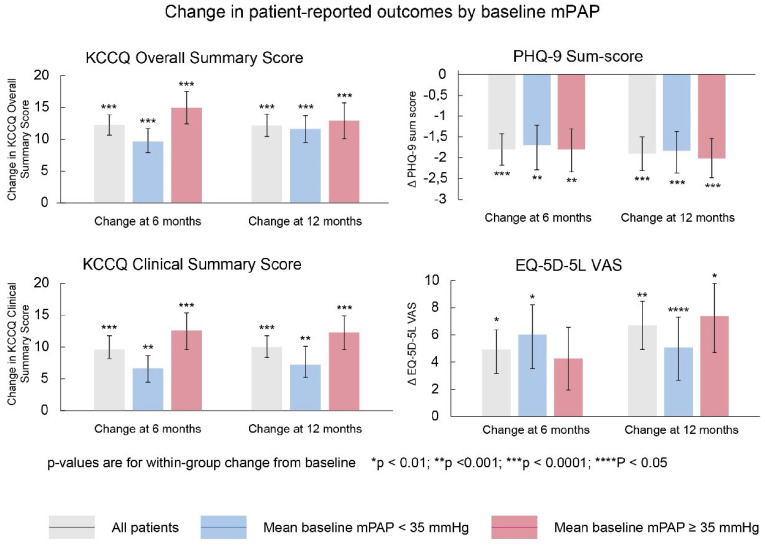
Changes in patient-reported outcomes by baseline mean pulmonary artery pressure (mPAP) as reported in the MEMS-HF study. CSS, clinical summary score; KCCQ, Kansas City Cardiomyopathy Questionnaire; OSS, overall summary score; PHQ-9, 9-item Patient Health Questionnaire; VAS, visual analogue scale. Reprinted from the European Journal of Heart Failure, Volume 22, Issue 10, Angermann et al., Pulmonary artery pressure-guided therapy in ambulatory patients with symptomatic heart failure: the CardioMEMS European Monitoring Study for Heart Failure (MEMS-HF), pages 1891–1901, with permission from Wiley [6].

PA pressure-guided HF management using the CardioMEMS HF system had been shown to be safe, reliable and effective in reducing HF hospitalizations in patients with chronic heart failure in NYHA class III only in the U.S. healthcare system. Considering the substantial differences in healthcare system organization, the question rose whether the findings could be extrapolated to Europe and be replicated outside the U.S. The CardioMEMS European Monitoring Study for Heart Failure (MEMS-HF) study was a prospective, non-randomized, multicenter study performed in sites from Germany, the Netherlands and Ireland and was published in 2020 [6]. The MEMS-HF study studied 234 NYHA class III patients with ≥1 HF admission in the past 12 months. The primary outcome was the reduction in HF hospitalizations. The primary safety endpoint was the freedom of DSRC and freedom of sensor failures at 1 year. In addition, quality of life was studied in more detail using the Kansas City Cardiomyopathy Questionnaire (KCCQ). The MEMS-HF was a post-marketing surveillance prospective study without randomization, in which patients were their own historical controls (using the event rate of the year pre-implant versus the year post implant of Cardio-MEMS). At 12 months’ follow-up, the rate of HF hospitalization was reduced by 62% with pulmonary artery pressure (PAP) monitoring. The freedom of DSRC at 1 year was 98.3% and freedom of sensor failures was 99.6%, which confirms the safety and durability of this technology (Figure 5). Considering quality of life, the KCCQ overall summary score improved from 47 at baseline to 60.5 (*p* < 0.001), which is considerable and clinically meaningful improvement (Figure 6) [6].

### 4.2. The CardioMEMS Post-Approval Study (PAS) 

**Figure 7:**
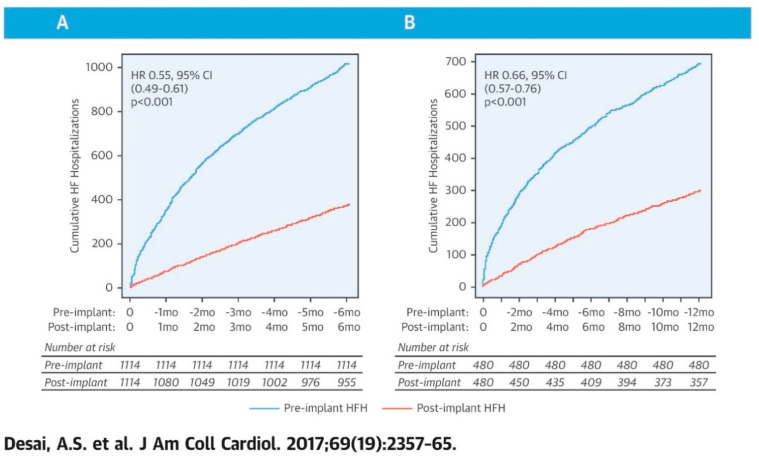
Heart failure hospitalization (HFH) rates before and after pulmonary artery pressure sensor implantation as reported in the CardioMEMS Post-Approval-Study. (**A**) 6 month cohort, (**B**) 12 month cohort. Reprinted from the Journal of the American College of Cardiology, Volume 69, Issue 19, Desai et al., Ambulatory Hemodynamic Monitoring Reduces Heart Failure Hospitalizations in “Real-World” Clinical Practice, pages 2357–2365, Copyright 2017, with permission from Elsevier [9].

The PAS was a multicenter prospective open-label study performed in 1200 chronic HF patients with a prior HF admission within 12 months from 104 sites from the United States and published its main results in 2020. Patients were selected irrespective of their HF subtype and ejection fraction percentage. Again, the hospitalization rates during the first 12 months post CardioMEMS implantation were compared to the hospitalization rates during the 12 months prior to CardioMEMS implantation. These patients had a mean age of 69 years and 62% were men. Background HF therapy (beta-blockers, RAS inhibitors, mineralocorticoid receptor antagonists (MRA) and ICD therapy) in HFrEF patients was contemporary and adequate [7]. The rate of HF hospitalization was significantly reduced with PA monitoring by the CardioMEMS HF system by 57% (HR 0.43; 95% CI 0.39–0.47, *p* < 0.0001) [7]. The freedom of device- or system-related complications at 1 year was 99.6% and freedom from sensor failure at 1 year was 99.9% (Figure 7) [7]. The rate of all-cause hospitalization was significantly lower at 1 year compared with the year before implantation as well. A striking result that was shown in this analysis is that the benefit of the CardioMEMS HF system is independent of ejection fraction and a significant reduction was shown in patients with heart failure with either reduced or preserved ejection fraction across all EF% ranges (EF < 40%; EF 40–50% and EF > 50%) [7]. No previous treatments or monitoring strategies have shown any benefit in patients with preserved EF. Monitoring PA pressures as surrogate of filling pressures and volume status is intuitive and clinically plausible to be effective in preventing overt clinical congestion in heart failure patients with preserved ejection fraction (HFpEF) [7].

### 4.3. The COAST Study 

In Europe, another post-marketing surveillance study is currently ongoing and some of the first-year results have been published in 2020. The CardioMEMS Post-Market Study (COAST) study aims to investigate the effectiveness of the CardioMEMS system in a post-marketing setting in Europe [8]. It includes NYHA class III chronic HF patients with HFrEF, heart failure with midrange ejection fraction (HFmrEF) or HFpEF, with at least 1 HF-related hospitalization in the last year. In 2020, the results from the first 100 patients from the United Kingdom (UK) included in the COAST study have been presented in an abstract at the 2020 ESC Heart Failure congress [8]. These patients had a mean age of 69 years, and 70% were men. In these first 100 patients, 1% of all patients developed a sensor- or procedure-related complication, and 1% had a sensor failure during the first 6 months of follow-up. A large reduction in the HF-related hospitalization rate was observed (2.05 HF-related hospitalizations/patient-year during the 6 months prior to CardioMEMS implantation vs. 0.33 HF-related hospitalizations/patient-year during the 6 months post CardioMEMS implantation) [8]. This initial interim analysis confirms the previously shown benefit of the CardioMEMS HF system in reducing HF hospitalization in Europe.

### 4.4. Real-World Studies

Several studies have examined the effectiveness of ambulatory hemodynamic monitoring by the CardioMEMS HF system in reducing HF hospitalization outside strict clinical trials or prospective study settings [9,10]. Desai et al. conducted a retrospective cohort study using 1114 patients from the U.S. Medicare claims database who had a CardioMEMS sensor implanted between 1 June 2014 and 31 December 2015 [9]. The investigators identified 1020 HF-related hospitalizations in the 1114 patients in the 6 months prior to receiving a CardioMEMS sensor implant and 381 HF hospitalizations, 139 deaths and 17 left ventricular assist devices or heart transplants (competing risks) in the 6 months after implant, which resulted in a 45% reduction in cumulative heart failure hospitalizations observed 6 months after PAP sensor implant versus in the 6 months prior to implantation (HR 0.55, 95% CI 0.49–0.61; *p* < 0.001) [9]. In those patients with complete follow-up data at 12 months (n = 480), results were similar with a 34% risk reduction (Figure 7) (HR 0.66, 95% CI 0.57–0.76). This Medicare analysis confirms the treatment benefit of the CardioMEMS HF system monitoring outside the clinical trial setting [9].

A second matched-cohort study from the Medicare beneficiaries’ claims database between 1 June 2014 and 31 March 2016 studied 1087 patients who received an implantable PA sensor and 1087 matched control patients [10]. Comparing the rates of HF hospitalization during the 12 months after CardioMEMS implantation between treatment and control group, the authors demonstrated a reduction of 24% (HR 0.76 95% CI 0.65–0.89). These Medicare analyses confirm the efficacy of the CardioMEMS HF system in the real world outside of clinical trial settings. Several smaller studies of single centers real-world experiences have reported similar effects and safety [14].

**Figure 8:**
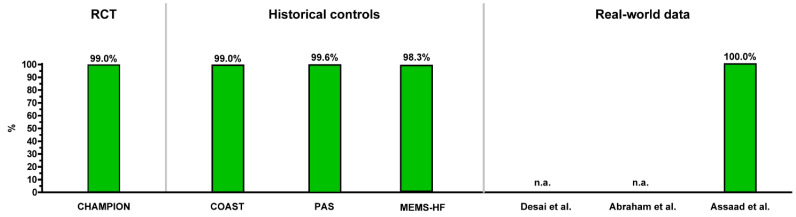
Overview of the safety (freedom of device- or system-related complications at 12 months) of the CardioMEMS HF system as reported in clinical studies.

**Figure 9:**
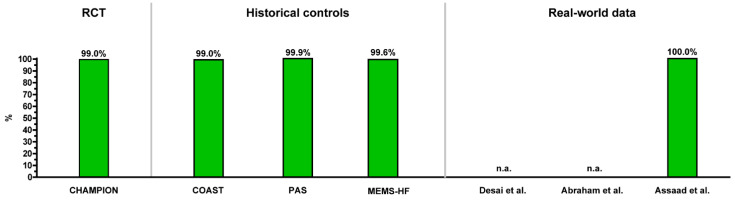
Overview of the reliability (freedom of sensor failures at 12 months) of the CardioMEMS HF system as reported in clinical studies.

Figure 8 and Figure 9 provide a summary overview of the safety and durability of the CardioMEMS HF system from all reported studies providing data on these issues. In general, the implant procedure is very safe with >98% freedom from DSRC, and the sensor is durable with a very low number of sensor failures at 1 year (>99% freedom of sensor failures).

**Figure 10:**
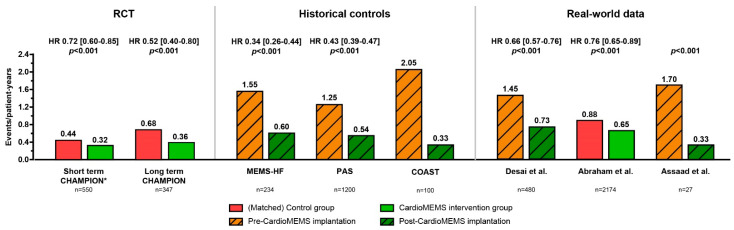
Overview of the clinical efficacy in reducing HF hospitalizations by the CardioMEMS HF system as reported in clinical studies. Legend: red and orange bars indicate either control group or the historical controls; green bars indicate the active CardioMEMS patient group; * Indicates event rate per patient per 6 months.

Figure 10 provides an overview of the clinical efficacy in reducing HF hospitalizations by the CardioMEMS HF monitoring system in all reported studies. In general, the treatment benefit of PA monitoring is evident and consistent from trial data to post-marketing surveillance data to real-world data. In addition, the benefit of CardioMEMS is shown across various types of HF patients and across EF% ranges.

An overview of the study characteristics and their results is shown in Table 1.

## 5. Ongoing CardioMEMS Projects

### 5.1. The GUIDE HF Trial 

In the United States, the GUIDE HF trial is currently enrolling and intends to test the efficacy of the CardioMEMS HF system in an expanded population, including patients outside the current indication of NYHA III but at risk of future HF events (ClinicalTrials.gov Identifier: NCT03387813) [15]. The trial will include 3600 patients with NYHA functional class II, III and IV and uses the criteria of either a prior HF hospitalization or a currently elevated NT-proBNP level above certain thresholds. Results are expected in 2021. The subgroup of NYHA class II HF patients is particularly interesting, as it has potential to significantly broaden the indications for CardioMEMS implantation.

### 5.2. The MONITOR HF Trial 

In the Netherlands, the MONITOR HF (Hemodynamic Monitoring In Heart Failure) trial started enrollment on 1 April 2019 and is currently enrolling patients in NYHA functional class III with 1 HF hospitalization within 12 months, defined as an admission for HF longer than 6 h and/or use of i.v. diuretics or emergency ward visit for HF resulting in i.v. diuretic therapy, independent of EF% (Clinical Trial Registration number NTR7672.) [16]. The MONITOR HF trial is a conditional reimbursement project to show the efficacy in a Western European setting against optimal contemporary heart failure therapy in a country with structured and dedicated HF care and outpatient clinics [16]. The MONITOR HF trial primarily investigates the experienced quality of life of HF patients in detail, as well as the number of HF hospitalizations and costs associated with remote monitoring.

## 6. Potential Use of Hybrid Constructions of Current Therapies with CardioMEMS Monitoring

Intuitively, the use of a remote hemodynamic monitoring tool provides unique opportunities for further research. For example, the effects of newer drugs like sacubitril-valsartan or sodium-glucose co-transporter-2 inhibitors prescribed in the outpatient clinic can be evaluated daily based on direct hemodynamic feedback, whereas this is conventionally assessed by judging patient status, laboratory values and echocardiography parameters which do not always accurately reflect hemodynamic status. Valvular heart disease, such as mitral regurgitation and aortic regurgitation, may generate volume overload and an increase in PA pressures without causing actual signs of congestion in the patient. In these cases, CardioMEMS may help assess the need for valvular intervention based on hemodynamic parameters, especially when drug interventions are unsuccessful in reverting pulmonary hypertension. After valvular intervention, CardioMEMS can accurately assess the hemodynamic effects of the intervention on a daily basis, which was not possible before. Furthermore, the PA pressure measurements can help titrate HF drugs as described in an example case report [17]. Finally, an attempt has been made to test the feasibility of combining remote hemodynamic monitoring in patients on LVAD support in the HEMO-VAD pilot study [18]. This study focussed on hemodynamic optimization, especially with diuretics, based upon PA pressures before surgery (to truly decongest the right ventricle and kidney in order to improve post-operative outcomes) and tests the feasibility of using CardioMEMS in the outpatient clinic phase of LVAD patients for assessment of volume status, arrhythmias and other VAD related complications [18,19,20]. A larger pivotal trial is underway in the U.S. to test the clinical use of CardioMEMS in optimization of LVAD support (INTELLECT2 trial, ClinicalTrials.gov Identifier: NCT03247829).

## 7. Future Perspectives

In 2021, several updates to the European Society of Cardiology and U.S. HF guidelines are expected, with a potential change in recommendations on CardioMEMS after the MEMS-HF and PAS data as well as real-world experiences. Additional research is needed to assess which patients are most likely to benefit from a PA sensor. In the upcoming years, the CardioMEMS HF system will most likely be upgraded with a CardioMEMS app, which is already being used in the United States. With increasing numbers of CardioMEMS implantations, over 20,000 in the USA in 2020, efforts need to be made to improve infrastructure for large-scale programs to maximize efficiency. A management-by-exception approach focusing on patients who are above their pressure threshold and reducing monitoring time in those patients within normal PA pressure range would be ideal. Currently, there are no structured treatment algorithms. Constructing such algorithms is a very complex issue, because they should provide therapy suggestions on an individual basis considering differences in treatment tolerance, blood pressure, renal function and many other comorbidities. Hospitals need to assess their telemonitoring platform or infrastructure in order to derive maximum benefit from ambulatory hemodynamic monitoring as well as reduce resource utilization and healthcare costs.

## 8. Conclusions

For patients with chronic heart failure in NYHA functional class III and a previous HF admission, the CardioMEMS HF system is a safe, reliable and proven clinically effective monitoring strategy to prevent HF hospitalizations. The benefit of remote monitoring using PA pressures has been shown across all EF ranges, including HFrEF and HFpEF. Monitoring of heart failure status by CardioMEMS is based on filling pressures instead of symptoms or bodyweight, and interventions mainly consist of remote optimization of diuretic or vasodilator doses. Clearly, by preventing HF hospitalizations using hemodynamic sensors such as CardioMEMS, future heart failure care can be optimized and healthcare resources better utilized for patients in need. In addition, costs of medical care can be reduced by preventing HF admissions via proactive early interventions.

## Figures and Tables

**Table 1:** Overview of study characteristics and patient baseline variables of CardioMEMS studies.

	Study Characteristics					Patient Baseline Variables *				
	First Author	Year of Publication	Country	Follow-Up Duration	Number of Patients	Mean Age	Male (%)	Background HF Therapy (%)		
								Beta-Blockers	RAS-i	MRA
**Randomized controlled trials**										
CHAMPION trial										
Open- access/extended period	Abraham ^4^	2016	USA	13 months	347 **	CM: 61.3 ± 13.0 Control: 61.8 ± 12.7	CM: 72 Control: 73	CM: 89 Control: 84	CM: 76 Control: 79	CM: 43 Control: 41
Randomized access period	Abraham ^5^	2011	USA	6 months	550	CM: 61.3 ± 13.0 Control: 61.8 ± 12.7	CM: 72 Control: 73	CM: 90 Control: 91	CM: 76 Control: 79	CM: 43 Control: 41
**Non-randomized controlled studies**										
MEMS-HF	Angermann ^6^	2020	DE/NL/IE	12 months	234	67.9 ± 10.7	78.2	88.9	85.5	72.2
PAS	Shavelle ^7^	2020	USA	12 months	1200	69 ± 12.0	62.3	88.1	56.9	44.1
COAST	Cowie ^8^	2020	UK	6 months	100	69 ± 12	70	NA	NA	NA
**Real-world studies**										
	Desai ^9^	2017	USA	6 months	1114	71.3 ± 10.8	63.8	NA	NA	NA
				12 months	480	71.4 ± 11.4	62.5	NA	NA	NA
	Abraham ^10^	2019	USA	12 months	2174	CM: 62.7 ± 10.2 Control: 72.9 ± 10.1	CM: 35.1 Control: 35.1	NA	NA	NA
	Assaad ^14^	2019	USA	6–18 months	27	67 ± 12	52	92.6	77.8	33.3

CHAMPION, CardioMEMS Heart Sensor Allows Monitoring of Pressure to Improve Outcomes in NYHA Class III Heart Failure Patients; COAST, CardioMEMS Post-Market Study; DE, Germany; IE, Ireland; NL, Netherlands; HF, heart failure; CM, CardioMEMS; MEMS-HF, CardioMEMS European Monitoring Study for Heart Failure; MRA, mineralocorticoid receptor antagonist; NA, not available; PAS, Post Approval Study; RAS-i, renin-angiotensin system inhibitors; UK, United Kingdom; USA, United States of America.* All studies were performed in patients suffering from chronic heart failure in NYHA class III across all ejection fraction ranges with a previous HF-related hospitalization. ** Primary efficacy analysis was performed in a subset of 347 from the 550 initially enrolled patients.

## Data Availability

Not applicable.
